# Epigenome-wide Association Studies and the Interpretation of Disease -Omics

**DOI:** 10.1371/journal.pgen.1006105

**Published:** 2016-06-23

**Authors:** Ewan Birney, George Davey Smith, John M. Greally

**Affiliations:** 1 European Bioinformatics Institute (EBI), Wellcome Trust Genome Campus, Hinxton, Cambridge, United Kingdom; 2 University of Bristol, School of Social and Community Medicine, Oakfield House, Oakfield Grove, United Kingdom; 3 Department of Genetics, Albert Einstein College of Medicine, Bronx, New York, United States of America; Stanford University School of Medicine, UNITED STATES

## Abstract

Epigenome-wide association studies represent one means of applying genome-wide assays to identify molecular events that could be associated with human phenotypes. The epigenome is especially intriguing as a target for study, as epigenetic regulatory processes are, by definition, heritable from parent to daughter cells and are found to have transcriptional regulatory properties. As such, the epigenome is an attractive candidate for mediating long-term responses to cellular stimuli, such as environmental effects modifying disease risk. Such epigenomic studies represent a broader category of disease -omics, which suffer from multiple problems in design and execution that severely limit their interpretability. Here we define many of the problems with current epigenomic studies and propose solutions that can be applied to allow this and other disease -omics studies to achieve their potential for generating valuable insights.

## The Epigenome-wide Association Study (EWAS)

“Epigenetic” processes have been defined in numerous ways: one example from Adrian Bird in 2007 uses the broad description “the structural adaptation of chromosomal regions so as to register, signal, or perpetuate altered activity states” [1]. Such activity states, when read out as transcription of genes, represent candidates for mediating between environmental, genetic, or stochastic factors and downstream phenotypes of the organism [2]. In theory, any perturbation of cellular homeostasis could be propagated through epigenetic mechanisms to cause a long-lasting phenotypic effect, especially if the perturbed cells are self-renewing stem/progenitor cells or long-lived, terminally differentiated cells. This logic has prompted an increasing number of studies [3] testing whether changes in patterns of epigenetic marks, almost always focused on DNA methylation, characterize individuals with a phenotype compared with control subjects. DNA methylation (5-methylcytosine [4]) is a covalent modification to DNA that can be faithfully propagated to daughter cells [5] and can exert transcriptional regulatory influences [6] and, therefore, has the necessary properties to mediate long-lasting perturbations of cellular states. When a pattern of changes of DNA methylation is found to occur repeatedly at specific loci, discriminating the phenotypically affected cases from control individuals, this is regarded as an indication that epigenetic perturbation has taken place that is associated, possibly causally, with the phenotype. This approach is described as an epigenome-wide association study (EWAS) [7], and takes its cue from the association of genetic variability with phenotypes in genome-wide association studies (GWAS).

## The EWAS as an Exemplar of Study Designs Problems in Disease -omics

The EWAS is representative of a number of high throughput molecular assays being used for associations with phenotypes of the organism (disease -omics) and is illustrative of some common problems with these approaches, as has been previously noted [7–9]. Epigenetic patterns may change during the lifetime of an individual [10,11]; therefore, epigenetic measurements represent part of the phenotype of the individual, akin to height or blood pressure.

In contrast, genetic measurements have two key properties. The first is that the vast majority of genetic loci stay constant over an individual’s lifetime (unless somatic mutations occur, as in cancer cells). This means that any observed association of genotype with phenotype cannot be attributed to phenotype-associated events changing the genotype. The second feature is that genetic variants can be assumed to be appropriately randomly assigned with respect to the characteristics of individuals [12]. When they are not randomly assigned, the strong signal of non-randomness across the entire genome is often identified as population stratification, amenable to correction using robust statistical techniques.

Any two measurements (physical or molecular characteristics) may be correlated within a population of people; the role of the epidemiologist is to ascertain *why* a particular correlation exists between two measurements. The first need is to get rid of spurious associations, including biased ascertainment when collecting the case and control individuals studied, the hidden presence of common factors underlying a supposed exposure, and the disease (a confounding effect) and reverse causation (in which the disease process influences the supposedly causal process, not the other way around [13–15]), see **Box 1**. The constancy and random assignment of genetic characteristics allow the case/control study design to succeed, permitting results to be interpreted as causal. In contrast, epigenetic measurements have all the same dangers as any other phenotypic measurement in a case/control design, including ascertainment issues and reverse causation effects.

**Box 1. Chance, Bias, and Confounding in Observational Studies**

Observational studies can suffer from a wide range of problems that lead to their findings being potentially misleading. We focus on biases that generate apparent associations that do not, in fact, exist in the population studied (“spurious associations”) and associations that are misleading indicators of underlying causal relationships.

**Spurious associations**

*Chance false positives and publication bias*: When a large number of associations can be examined within a dataset, it is inevitable that, by chance, some will appear to have reasonable statistical evidence attached to them. This leads to the phenomenon of multiple testing linked to publication bias, the tendency of “statistically significant” findings being preferentially published, increasing the chances of false positive results ending up in the literature. This sequence of events is a contributory factor for the very poor replication record for published candidate gene studies, whereas in the GWAS era, robust methods were applied to correct for multiple testing.

*Ascertainment and other selection biases*: The ascertainment of cases of disease in case-control studies can lead to a non-random proportion of all possible cases being included in a study, with factors related to ascertainment appearing to be risk factors for the disease, even though they are not associated with the disease within the source population. Other forms of selection bias can lead to the same situation.

**Reliable but non-causal associations**

*Confounding*: An underlying factor can influence both the studied exposure and the apparent outcome, generating a non-causal but reliably observed association. For example, the oft-used example of the confounded association between yellow fingers and lung cancer—both caused by cigarette smoking—would lead to a real, but non-causal association between the two. Due to inevitable measurement error in characterizing the confounding factor, and the likelihood that there are unmeasured confounders, conventional statistical adjustment approaches have a well-documented limited ability to remove confounding adequately in observational studies.

*Reverse causation*: A special case of confounding is when the disease process influences the exposure, rather than vice versa. This can occur well before the disease becomes evident, thus prospective studies with assessment of risk factors before the observed development of disease are not immune to this problem.

While spurious associations always need to be avoided, reliable but non-causal associations can still be useful as predictive, prognostic, or diagnostic indicators. For example, if cellular heterogeneity in the tissue studied contributes to the age-prediction utility of DNA methylation data [17], removing the cell subtype influences analytically would be counterproductive for the use of DNA methylation as a biomarker in this case.

## Problems Interpreting EWAS Results

In parallel to these epidemiological issues, there is a further layer of complexity in the interpretation of the results of the epigenomic assays. We now appreciate that reported DNA methylation differences between individuals may reflect something other than epigenetic changes in a specific cell type. One major focus has been on the potential for cell subtype proportional heterogeneity to influence the DNA methylation patterns observed in pools of cells. This was highlighted by Houseman and colleagues in a study showing that altering the proportions of purified cells in a mixture generates different DNA methylation profiles, reflecting the distinctive DNA methylation patterns of each cell type present [16]. It was subsequently shown that cell subtype effects accounted for a major proportion of the epigenetic changes associated with ageing in a re-analysis of five studies of peripheral blood leukocytes [17]. These findings of the influence of cell subtype heterogeneity prompted the development of new analytical approaches to account for this effect [16,18]. Even when cells are “purified” using cell surface markers, we find evidence for further cell subtypes with distinctive DNA methylation patterns [19]. It is, therefore, likely that even when using purification techniques, a pool of cells is composed of multiple epigenomes, generating what we refer to as a “meta-epigenome” [19].

Even after the most careful attempts to address the influence of cell subcomposition [20] or when histologically homogeneous cells are studied [21], the outcome of the EWAS generally identifies only modest changes in DNA methylation. As DNA methylation genome-wide is very bimodal, with the majority of loci in a diploid organism methylated on neither (0%) or both (100%) of the alleles present, a change of DNA methylation of, for example, 20% has to represent a changed proportion of alleles with the DNA methylation mark, in turn indicating a cellular mosaicism for the epigenetic changes associated with the phenotype. With the development of single-cell techniques to study DNA methylation [22,23], these mosaic events will be able to be confirmed experimentally. The small degree of change represents the strongest current justification for DNA methylation to be used as the primary molecular assay in EWAS, as other assays (such as those based on chromatin immunoprecipitation) have only rarely been demonstrated to have the quantitative capacity required to detect events occurring in only a subset of alleles tested [24]. From a practical perspective, it is also more challenging to collect samples for chromatin-based assays from human subjects, another reason for gravitating to the study of DNA methylation.

Similar limited degrees of change of DNA methylation are also appreciated to result from transcription through a genomic region [25,26]. A change in DNA methylation in a region that is polymorphically transcribed between individuals may, therefore, generate DNA methylation changes that are due to (and not causative of) the transcriptional changes. Of even greater concern is the influence of DNA sequence polymorphism. This influence appears to be very powerful, estimated to account for 22% to 80% of the variability (degree of change or proportion of loci) in DNA methylation between individuals [27–29]. In germline genetic studies, the complications due to variability of ancestry can be addressed through population stratification approaches and knowledge of linkage disequilibrium patterns, but no comparable strategies exist for epigenomic studies. The degree of change of DNA methylation associated with sequence polymorphism is limited, again indicating a mosaic cellular response to this influence.

As EWAS have generally been only rarely performed with concurrent genotyping of the same individuals [21,30] or transcriptional studies of the same cells [29], we have no way of knowing whether the positive results of EWAS to date are testing the starting hypothesis that genuine epigenetic changes occur within a subset of cells in the population. Instead, the results may be due to residual meta-epigenomic effects of cell subtypes or attributable to untested influences of genomic or transcriptomic variability. This being the case, and with similar caveats affecting transcriptomic studies, no EWAS to date can be said to be fully interpretable.

## How to Strengthen EWAS and Other Disease -omics Study Designs

**Fig 1:**
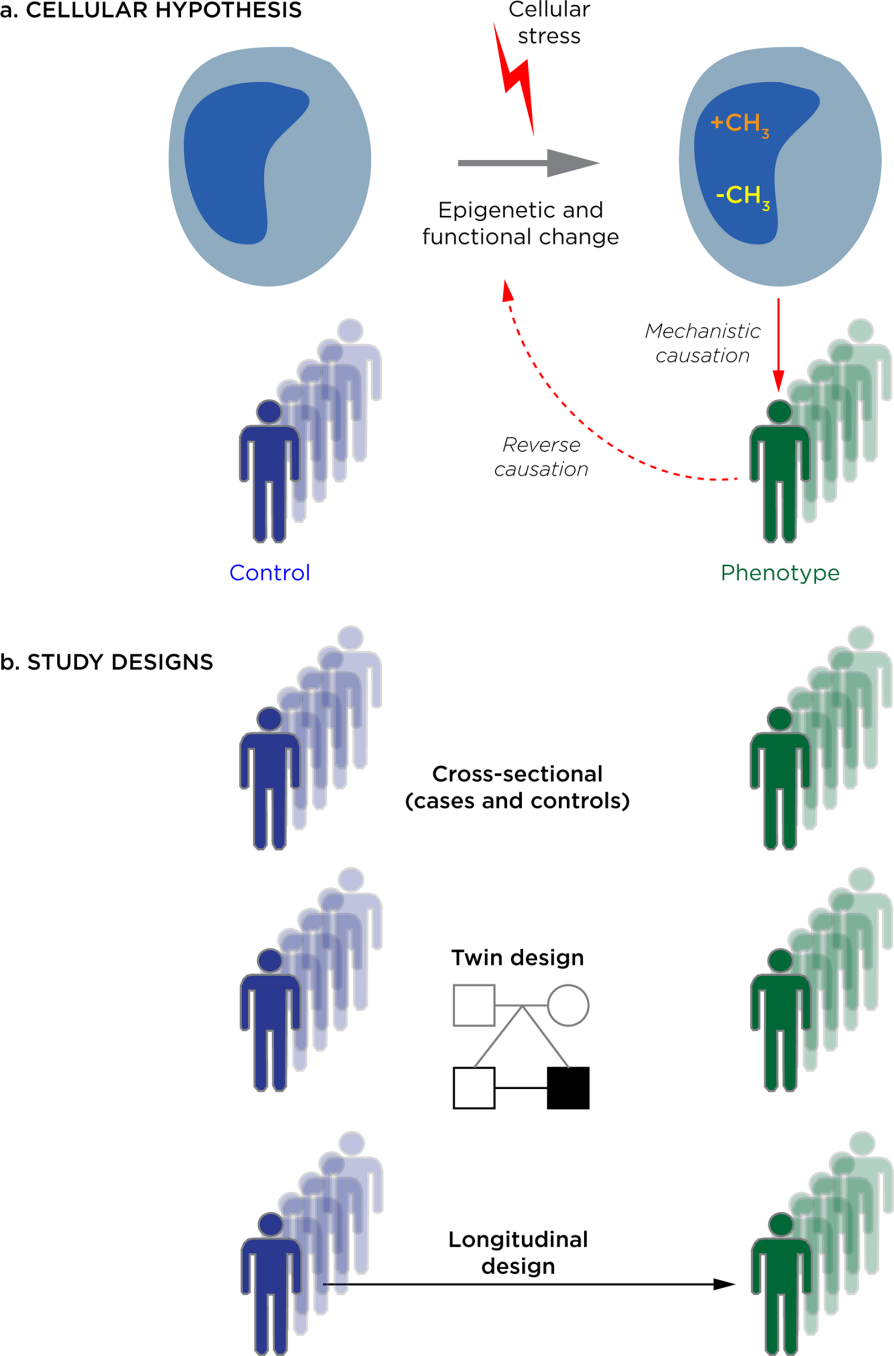
An overview of considerations in designing and interpreting epigenome-wide association studies (EWAS). In (a) we explicitly define the cellular hypothesis being tested in EWAS: that there are changes in epigenetic mediators of transcriptional regulation (denoted by gains or losses of methyl groups) that distinguishes a canonical cell type in individuals with a phenotype (green) from those without the phenotype (blue). The EWAS is frequently performed to address the idea that the epigenetic dysregulation is occurring as a response to a cellular exposure or stress. In a situation of reverse causation, the reason the epigenetic change is observed in association with a phenotype is because the phenotype induces the epigenetic change, rather than the other way around. In (b) we describe three study designs: the typical cross-sectional design comparing individuals with and without the phenotype of interest, and two designs that reduce the effects of genetic polymorphism, which are studies of monozygotic twins discordant for a phenotype, and longitudinal studies of people before and after they develop the phenotype.

The key to improving the interpretability of epigenetic studies is their optimal planning at the outset. We illustrate some of the issues involved in designing and executing these studies in **Fig 1**, such as the cellular hypothesis being tested and the cohorts that could be compared. While different study designs will suit different questions, it is unfortunate that the easiest study design to execute, the cross-sectional case/control approach, is generally a suboptimal choice. This is due in part to the ascertainment issues referred to earlier and in part the possibility of reverse causation, in which the epigenomes of cells tested are influenced by (rather than part of the causal process leading to) the disease, as shown recently in a study of body mass index [31]. It is important to stress that this lack of interpretability cannot be fixed with increased sample size or choice of cell type; it is inherent to the design of the study.

A longitudinal design is advantageous for both biomarker and mechanistic insights. Longitudinal sampling of cells from an individual allowing studies prior and subsequent to disease onset allows the identification of the epigenetic changes that precede the development of the overt phenotype. This design overcomes some of the concerns raised earlier but, obviously, requires the foresight to capture informative samples from individuals who go on to develop diseases. While this seems like a major challenge, it should be recognized that epidemiologists have been developing and managing cohorts with this in mind for many decades. Banked biological material is most likely to be blood, allowing opportunities for studying the wide range of phenotypes mediated by leukocytes. In addition, one might be able to see changes in white blood cell epigenetic profiles as potential markers of exposures or predictors of disease risk or prognosis. If the epigenetic measurement is robust as a biomarker, it does not matter whether this is due to epigenetic modifications in the cells tested or reflective of cell subtype, transcriptional, or DNA sequence effects on DNA methylation. If the goal is to understand causal mechanisms, however, these confounding influences need to be taken into account.

To account for confounding biological influences, transcriptional studies of the same cells need to be performed to understand the bidirectional interactions of transcriptional and epigenetic processes. Genotyping has to be used to define the loci that are variable in response to DNA sequence differences, which may be facilitated by extracting DNA sequence variant information when bisulphite sequencing is performed to study DNA methylation [32]. The use of purified or histologically identical cells is not enough to eliminate cell subtype effects [19] but is likely to diminish this influence. Any means of quantifying cell subtype composition using cell biology approaches or analyses of molecular characteristics (such as *CellMix* [33]) should be employed to measure the cell subtype proportions. All of these measures are in addition to those normally applied in projects involving complex molecular assays, which require the systematic collection of experimental metadata and quality information to test whether the experiments themselves have contributed to the variability observed. We summarize these recommendations in **Box 2**.

**Box 2. How to Improve the Interpretability of EWAS Data**

We provide here a checklist of ways to improve EWAS studies:

- Start with a clear hypothesis—do you seek to understand the mechanism of the disease or phenotype, in which case a mediating cell type with high purity should be studied, or do you want to identify a biomarker (of exposure or of predictive/prognostic value), in which case a surrogate, accessible cell type may be used?
- Carefully consider whether your study design can answer this hypothesis. Note that using a case/control study design will, by definition, have a complex ascertainment following disease onset and will not easily discover biomarkers or causal mechanisms. This is not a property of sample size, rather a property of ascertainment.
- Purify the cell type as much as possible, and use whatever means available to understand the cell subtype heterogeneity present in the tested samples.
- Perform transcriptomic studies on the same cells tested for epigenetic changes and genotyping of the same individuals. This allows a number of causes and consequences of changes of epigenetic regulators to be interpreted.
- Analytically, account for any epigenetic variability that is due to cell subtype, transcriptional or sequence variability, as well as any identifiable technical factors occurring during the experiments and captured as metadata.
- When attempting to understand the mechanistic role of epigenetic dysregulation in the phenotype, interpret the degree of change of DNA methylation. If modest, and therefore involving a mosaic subset of cells, how does this contribute to mechanistic understanding?

Analytically, insights into DNA sequence variants upon DNA methylation (methylation quantitative trait loci, mQTLs [34]) for the cell type studied will allow approaches to be developed to account for this major influence upon the epigenome. One particular approach, two-step mendelian randomization, is being applied in prospective and case/control EWAS, building on the non-modifiable nature of germline genetic variation to provide causal anchors within a causal inference setting [35,36]. This and other new methodological approaches to integrate epigenetic, transcriptomic, and genotypic information will require the involvement of analytical specialists to work with these rich but complex datasets.

## Conclusions

We focus here on the EWAS, not only because of the general lessons it allows when designing other disease -omics studies but also because we now have insights into biological influences that can influence the epigenome. Furthermore, there is the exciting possibility that well-designed studies of the epigenome can generate substantial new insights into disease mechanisms and valuable biomarkers. To realize this potential for epigenomic studies and other disease -omics, many aspects of current approaches need to be reconsidered. We provide specific recommendations for study design with the goal of prompting a discussion about how to improve the interpretability of the results when these studies are completed.
